# Algorithms to estimate the lower bounds of recombination with or without recurrent mutations

**DOI:** 10.1186/1471-2164-9-S1-S24

**Published:** 2008-03-20

**Authors:** Xiaoming Liu, Yun-Xin Fu

**Affiliations:** 1 Human Genetics Center, School of Public Health, University of Texas at Houston, Houston, Texas 77030, USA

## Abstract

**Background:**

An important method to quantify the effects of recombination on populations is to estimate the minimum number of recombination events, *R_min_*, in the history of a DNA sample. People have focused on estimating the lower bound of *R_min_*, because it is also a valid lower bound for the true number of recombination events occurred. Current approaches for estimating the lower bound are under the assumption of the infinite site model and do not allow for recurrent mutations. However, recurrent mutations are relatively common in genes with high mutation rates or mutation hot-spots, such as those in the genomes of bacteria or viruses.

**Results:**

In this paper two new algorithms were proposed for estimating the lower bound of *R_min_* under the infinite site model. Their performances were compared to other bounds currently in use. The new lower bounds were further extended to allow for recurrent mutations. Application of these methods were demonstrated with two haplotype data sets.

**Conclusions:**

These new algorithms would help to obtain a better estimation of the lower bound of *R_min_* under the infinite site model. After extension to allow for recurrent mutations, they can produce robust estimations with the existence of high mutation rate or mutation hot-spots. They can also be used to show different combinations of recurrent mutations and recombinations that can produce the same polymorphic pattern in the sample.

## Background

### Introduction

Recombination is an important mechanism for shaping genetic polymorphism. Estimating the effects of recombination on polymorphism plays important roles in population genetics [1]. One direct measure of the amount of recombination is the minimum number of recombination events in the history of a sample. However, not all recombination events occurred on the genealogy of a sample can be detected [2]. We can only estimate the minimum number of recombination events, *R_min_*, which can be interpreted as, at least how many recombination events occurred in the history of a sample. Estimating *R_min_* is by no means an easy task, so that most of the previous work focused on the lower bound of *R_min_*, which is also a valid lower bound of the true number of recombination events occurred.

The seminal work of Hudson and Kaplan [3] introduced a lower bound on such minimum number, *R_m_*, which is based on the four-gamete tests under the infinite site model. For each pair of polymorphic sites, if there are four distinctive haplotypes (four-gamete), the data is said to be inconsistent and at least one recombination must occur in that interval. Assuming all overlapping four-gamete intervals are caused by the same recombination event, *R_m_* is obtained by counting the total number of non-overlapping four-gamete intervals. Of course, there is a large chance this assumption does not hold. So *R_m_* can be quite conservative. Hein and his colleagues [4-6] used dynamic programming to estimate *R_min_*, which guarantees that the true minimum number can be found. Nevertheless, the computational intensiveness prevents its application to a moderate number of sequences. Recently, Myers and Griffiths [7] introduced a new method based on combining recombination bounds of local regions (local bounds) to estimate a global composite bound of the sample. This method shows a large improvement over *R_m_* while it is applicable to moderate to large data sets. Further improvements of local bounds have also been suggested by Song et al. [8], Lyngsø et al. [9], Song et al. [10] and Bafna and Bansal [11], which will be discussed in more detail in the next subsection.

This paper proposes two new improved lower bounds under the infinite site model and their extension to allow for recurrent mutations. The performances of these lower bounds are compared to those of other lower and upper bounds via simulation. Two real data sets are analyzed to demonstrate the application of these new bounds. Approximation algorithms for the bounds are also discussed in this paper.

### Previous work on local bound

Myers and Griffiths [7] introduced two new local bounds under the infinite site model and one method to combine them into a global bound. The basic idea is that, since the algorithms available perform better on a sample of sequences with small number of polymorphic loci than on that with large number of loci, we can cut the sequences into small segments, estimate the lower bound of each segment and then combine them into a global bound for the whole sequences. It is easy to understand that a better local bound would improve the estimation of *R_min_* when combined. In this subsection we summary the previous work on local bounds, and in next section we propose our new algorithms on improving and extending the estimation of local bounds.

To discuss the problem of local bound formally, let us assume a matrix *M* with *n* rows and *m* columns. Each row represents a sequence or haplotype and each column represents a polymorphic site. We further assume that there are only two allele types, say 0 and 1, at each polymorphic site, which is the most common case for SNPs. Given a set of sequences, an allele type is called mutation if that type has only one copy in the set; a polymorphic site is called informative if each allele type of this site has more than one copy in the set. A local bound is a lower bound of the number of recombination events occurred in the unknown history of the sequences in *M.*

The local bound *R_h_* by Myers and Griffiths [7] is called a haplotype bound. It is based on the observation of the haplotype number change on an ancestral recombination graph (ARG) [12]. The original algorithm Myers and Griffiths [7] provided is a heuristic search algorithm. Song et al. [8] described an algorithm based on an integer linear programming to compute the optimal *R_h_-* Bafna and Bansal [11] suggested another local bound estimator, *R_g_,* which is an approximation of *R_h_* calculated with a greedy search algorithm. The local bound *R_s_* by Myers and Griffiths [7] is estimated through tracing the history of the sample, which is similar to that of coalescent simulation. However, the specific topology and length of the branch are ignored. Myers and Griffiths [7] showed in their paper *R_s_ ≥ R_h_ ≥ R_m_* when their global bounds were compared.

**Figure 1:**
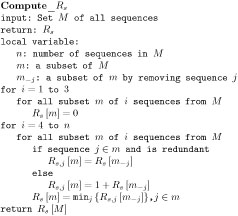
Bafna and Bansal's algorithm for *R_s_*.

**Figure 2:**
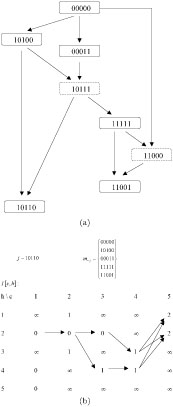
An example of recombination intermediates (a) and computation of *I*_j_ [*m*_*−j*_] (b).

Bafna and Bansal [11] proposed a faster algorithm for computing *R_s_* (Figure 1), which views the history of the sequences prospective in time other than retrospective in time as the original algorithm. Given a history, there is a particular order of sequences associated with the history (see Figure 2 (a) for an example). Assume the order is *r_1_,r_2_,r_3_*, …, where *r_j_* represents a sequence with rank *j,* then all *r*_*i*_ with *i < j* are potential ancestor sequences of *r_j_.* Let set *m* = {*r_1_, r_2_, … , r_j_*} and *m_−j_ = {r_1_, r_2_, … , r_j−1_}*. Regarding the informative sites of *m* only (that is, ignoring mutations), if *r_j_* is identical to any sequences in *m_−j_* (i.e. redundant), *r_j_* can be derived from *m_−j_* via only mutations; otherwise at least one recombination event is needed. The algorithm adds sequences one by one following a particular order. Whenever a new sequence added is not redundant, the algorithm counts one recombination. After all possible orders of sequences are examined, the smallest count of an order is regarded as *R_s_.* Of course, when a non-redundant sequence added, counting only one recombination event is quite conservative. Lyngsø et al. [9] suggested a branch and bound search of the exact position of crossovers on the ancestral sequence to produce a true ARG. Song et al. [10] further extended the method to allow for gene conversion events. Alternatively, Bafna and Bansal [11] introduced an algorithm for computing the minimum number of recombination events, *I_j_*[*m_−j_*]*,* needed to obtain a recombinant *j* given a set, *m_−j_,* of its possible ancestors. The the crucial part of the algorithm is computing the recurrence

$$I[c,h]=\{\begin{array}{ll} \infty & \text{if }j[c]\neq h[c]\\ 0 & \text{if }j[c]=h[c]\;\text{and}\;c=1\\ I_{min} & \text{if }j[c]=h[c]\;\text{and}\;c>1 \end{array}$$

where

$$I_{min}=\min\left\{I[c-1,h],\underset{h^{\prime }\neq h}{\min}\left\{1+I[c-1,h']\right\}\right\},$$

*h* [*c*] represents the allele type of sequence *h* at site *c* and *j*[*c*] ≠ *h*[*c*] is true only when the two allele types are not missing and different to each other. *I* [*c, h*] can be interpreted the minimum number of recombinations needed to explain the first *c* informative sites of sequence *j* with *h* [*c*] as the parent of *j* [*c*]. Then

$$I_{j}\left[m_{-j}\right]=\min_{h}\left\{I\left[s,h\right]\right\},h\in m_{-j},$$

where *s* is the number of informative sites of sequences in set *m* = *m_-j_* ∪ *j*.

*I*[*m_−j_*] can be larger than one if more than one recombination is needed to produce sequence *j.* In such situations, some recombination products are not presented in the sample and are called recombination intermediates [11]. Figure 2(a) presents a genealogy of the sequences with their top-down vertical positions corresponding to a particular (adding) order of the sequences, where 0 and 1 represent the two alleles on each site. The sequences in the boxes with solid lines are presented in the sample while those in the boxes with dashed lines are recombination intermediates. Figure 2(b) is an example showing the computation of *I_j_* [*m_−j_*] with *j* = 10110 and *m_−j_* = {00000, 10100, 00011, 11111, 11001} as in Figure 2(a), where arrows show how the final value two is obtained.

In Bafna and Bansal's [11] prospective algorithm for *R_s_* (Figure 1), each time when a recombinant is added, one is added to the count of recombination events. At first glance, we can just replace one by *I_j_* [*m_−j_*]. However, since the recombinant intermediates are unknown, it is possible some of them are parents of other sequences in the sample. So that the same recombination events may be counted more than once when adding these daughter sequences, which violates the definition of lower bound. Although this quantity is no longer a lower bound, it is still informative. Song et al. [8] named it *R_u_*, as the upper bound of *R_min_*, which can be interpreted as at least how many recombination events are enough to obtain the sample. To avoid counting any recombination intermediate more than once, Bafna and Bansal [11] introduced the concepts of *direct witness* and *indirect witness* of a recombination event. A sequence is a direct witness if it is the direct product of a recombination, i.e. recombinant. A sequence is an indirect witness if it is derived from a recombinant via mutations. For example, in Figure 2(a) 11111 is an indirect witness and 10110 is a direct witness. Based on that they proposed the algorithm of *R_I_* which adds the minimum number of recombination intermediates of only one direct witness to the total count of recombination events, which avoids multiple counting of recombination intermediates and make *R_I_* a valid lower bound [11]. The original algorithms for *R_u_* and *R_I_* approximate the quantities over all possible orders of sequences [8,11]. Algorithms A.1 and A.2 in Appendices A show the corresponding *R_u_* and *R_I_* for a particular order of sequences, which is useful when only a small set of orders need to be examined. Here is an example to compute *R_u_* and *R_I_.* In Figure 2(a) the unobserved recombinant intermediate 10111 produces both 11111 and 10110 in the sample. Suppose the order of the sequences is 00000, 10100, 00011, 11111, 11001 and 10110 according to their vertical positions in the figure. With this particular order, we obtain *R_u_ =* 5, because other than the two recombinations counted for 11001 and one for 11111, two more recombination events are needed to explain 10110 (Figure 2(b)), which can also be regarded as an additional count of the recombinant intermediate 10111. For the particular order of sequences in Figure 2(a), *R_I_ =* 3.

## Results and discussion

### Improved lower bounds under the infinite site model

**Figure 3:**
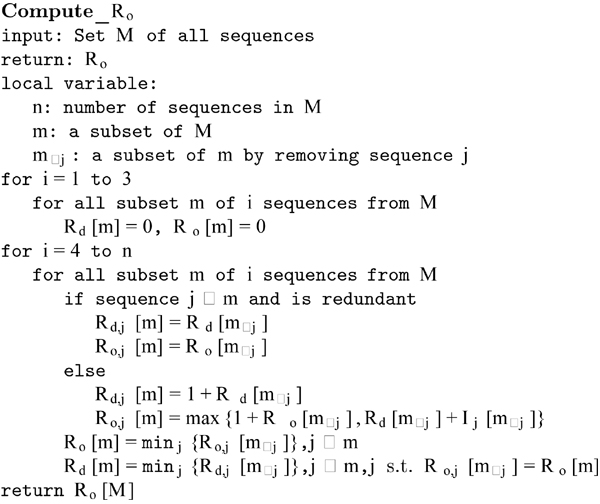
An algorithm for computing *R_o_*.

In Bafna and Bansal [11]'s original algorithm for *R_I_,* the counting of the number direct witnesses and the counting of total number of recombination are independent to each other and may not correspond to the same order of the sequences. However, a particular order of sequence is associated to an ARG, which is very informative itself. Here we propose a modified lower bound called *R_o_* to overcome this disadvantage. The “o” in *R_o_* stands for order, which counts the number direct witnesses and the total number of recombinations depending on the same order of sequences. The detailed steps are presented in Figure 3 (and Algorithm A.2 in Appendices A for a fixed order of sequences).

It is easy to understand that all the difficulties of counting the minimum number of recombination events are due to the fact that all recombination intermediates are unknown. Ideally, if in the process of computing *R_s_* or *R_I_,* when adding a recombinant *j* to *m_−j_*, we also add its recombinant intermediates leading to *j,* the true *R_min_* can be obtained. It seems straightforward to recover the recombinant intermediates simply by tracing the “path” leading to the final *I_j_* [*m_−j_*], just as the arrows displayed in Figure 2(b). However, this strategy could be very inefficient because typically there will be multiple paths to the same *I_j_* [*m—j*] so that many possible recombination intermediates. Although some of the intermediates may be redundant, the possible number of distinctive intermediates may still be large. In the case of Figure 2(b), four different paths lead to the same final value of two, each with two break points. There are a total of three distinctive intermediates, 1011*, ***10 and **110, where * represents a site that is not the ancestor of the corresponding site of sequence *j,* so that its allele type is not of interest. To find the final lower bound, one needs to store all possible combinations of recombinant intermediates as augmented sequences in a set, say *m*′, at each step of adding a recombinant. Each *m*′ will be used as the possible parent sequences when adding the next recombinant. The number of *m*′ can grow exponentially at each step of adding a recombinant, so does the computational time. Alternatively, we can make a compromise by adding some, but not all, recombinant intermediates.

**Figure 4:**
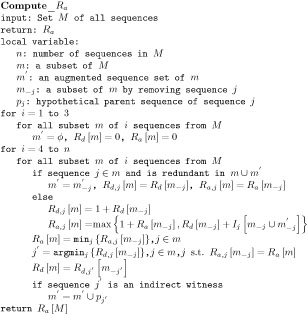
An algorithm for computing *R_a_*.

One immediate candidate is the hypothetical parent sequence of an indirect witness. If only one new mutation is introduced to *m* from an indirect witness *j,* a hypothetical parent sequence of *j* is formed by replacing the mutant allele on the mutation site with the “wild-type” allele presented in all sequences in *m_−j_*. For example, in Figure 2(a) the hypothetical parent sequence of 11111 is 10111. If more than one new mutation is presented in *j*, a hypothetical parent sequence of *j* is formed by replacing all the mutant alleles with a missing data '?', which can be either the mutant allele or the “wild-type” allele. Based on this, here we propose another improvement over *R_I_*, which is called *R_a_.* The “a” in *R_a_* stands for augmentation, which augments the hypothetical parent sequences of indirect witnesses into the sample during the process. The detailed steps are presented in Figure 4. The algorithm (Algorithm A.3) and a proof (as a valid lower bound) for *R_a_* with a particular order of sequences are given in Appendices A and B, respectively. As to the example in Figure 2(a), Algorithm A.3 recovers the recombination intermediate 10111 and *R_a_ =* 4, which equals to the true number of recombination events presented.

### Extension to allow for recurrent mutations

The lower bounds developed under the infinite site model assume all polymorphic inconsistencies are caused by recombination. However, recurrent mutations, commonly observed on mutation hot-spots, also can cause inconsistency. There is a difference though. The former is more likely to affect a long range of sites because a segment of DNA was involved in recombination. On the other hand, recurrent mutation occurs one site at a time, so that it is unlikely to observe inconsistent sites clustering together in a long range. This difference has been used to detect recombination and find breakpoints [1,13]. However, the difference is by no means clear-cut, especially when SNP data other than sequence data is used, some information of the spacial inconsistent pattern is lost. As a result, it is difficult to distinguish recombination from recurrent mutations. Nevertheless, it is informative to give a conservative estimation of the upper and lower bounds of *R_min_* with the consideration of recurrent mutations.

This can be done by extending *I* [*c, h*], which can be regarded as the minimum cost if *h* [*c*] is the parent of *j* [*c*]. In its recurrence, if *j *[*c*]* ≠ h*[*c*]*, I* [*c*, *h*]* =* ∞. This is due to the fact that if *j* [*c*] ≠ *h* [*c*] and *h *[*c*] is the parent of *j *[*c*], then *i *[*c*] must be produced by a recurrent mutation on that site, which is not allowed under the infinite site model. So that, the computation of *I* [*c*, *h*] is a dynamic programming process which assigns a cost of ∞ to a recurrent mutation and 1 to a recombination, and minimizes the cost of all informative sites of sequence *j.* This minimum cost is also the minimum number of recombination events, since only recombination is allowed and each costs 1.

To allow for recurrent mutations, we can simply assign a cost other than ∞ to it. Assume the costs of recombination and recurrent mutation are *c_r_* and *c_m_,* respectively, then replace *I* [*c*, *h*] with *I*′ [*c, h*] as

$$I^{\prime }[c,h]=\{\begin{array}{ll} 0 & \text{if }j[c]=h[c]\;\text{and}\;c=1\\ c_{m} & \text{if }j[c]\neq h[c]\;\text{and}\;c=1\\ {I^{\prime }}_{min} & \text{if }j[c]=h[c]\;\text{and}\;c>1\\ {I^{\prime }}_{min}+c_{m} & \text{if }j[c]\neq h[c]\;\text{and}\;c>1 \end{array}$$

where

$${I^{\prime }}_{min}=\min\left\{I^{\prime }[c-1,h],\underset{h^{\prime }\neq h}{\min}\left\{c_{r}+I^{\prime }\left[c-1,h^{\prime }\right]\right\}\right\}.$$

Again we minimize the total costs of all sites of sequence *j.* Then *I_j_*[*m_−j_*] records the number of recombinations (along with the number of recurrent mutations) that gives the minimum *I′ *[*s, h*] of all *h* ∈ *m_−j_.* Song et al. [10] used a similar approach to incorporate gene conversion event into their search algorithm for the lower and upper bounds of *R_min_*.

This simple extension can be easily applied to *R_I_, R_o,_ R_a_* and *R_u_* since they all use the quantity *I_j_* [*m_−j_*]. With this extension, they will be presented as *R_fi_* (*c_m_*, *c_r_*), *R_fo_* (*c_m_*, *c_r_*), *R_fa_* (*c_m_*, *c_r_*) and *R_fu_* (*c_m_*, *c_r_*). We can allow different number of continuous recurrent mutations with different combinations of *c_r_* and *c_m_*. For example, the procedure with *c_m_* = 3 and *c_r_* = 2 will prefer one recurrent mutation than a double recombination crossover (gene conversion) at a single inconsistent site, but will prefer a double crossover than two or more recurrent mutations at continuous sites. So that *c_m_* = 3 and *c_r_* = 2 can be used as a conservative lower bound of *R_min_* with the assumption that a small number of mutation hot-spots are present and distributed evenly on the sequence. If per bp recombination rate (*r*) and mutation rate (*μ*) are known, the procedure with *c_m_* = lg *μ* and *c_r_* = lg*r* will find the maximum likelihood estimation of the number of recombination events. We need to be careful about the interpretation of these extended bounds. They are just conservative estimations of the corresponding lower or upper bounds under the infinite site model.

Another usage of this extension is to show what combination of recurrent mutations and recombinations can produce the same observed inconsistency. The lower and upper bounds under the infinite site model are of one extreme, which show the minimum number of recombination events required to produce the pattern if there is no recurrent mutations. The maximum parsimony tree method used in the phylogenetic study is of another extreme, which shows the minimum number of recurrent mutations needed to produce the pattern if there is no recombination. Because a byproduct of *R_fo_* (*c_m_, c_r_*) and *R_fu_* (*c_m_, c_r_*) is the fully determined number of recurrent mutations associated with a particular order, which can be used to show different combinations of recurrent mutations and recombinations that can produce the same polymorphic pattern. We will show this usage in **Examples**.

### Performance comparison

To compare the performances of these lower bounds, we conducted coalescent simulations to generate samples and then obtained estimations from the bounds. To simulate a sample, we assumed the values of two crucial population parameters, population mutation rate *θ =* 4*N μ* and population recombination rate *ρ = 4Nr,* where *N* is the effective population size and *μ* and *r* are mutation rate and recombination rate per gene per generation, respectively. With different combinations of *θ (θ=5,* 10, 20, 50, 100) and *ρ (ρ=0,* 1, 5, 10, 20, 50, 100), 10,000 independent samples were simulated with sample size *n* = 10. The ms program [14] was used to conduct the simulation.

To study the performances of the local bounds under the finite site model, we used the ms program to simulate gene genealogies and then used the Seq-Gen program [15] to simulate DNA sequences with 2501bp in length given these gene genealogies. For each simulation a Kimura 2-parameter model [16] was used with a large transition to transversion ratio, which made each site only had two alleles so that the bounds developed under the infinite site model can also be computed. For each combination of *θ* and *ρ,* 10,000 samples were simulated.

**Figure 5:**
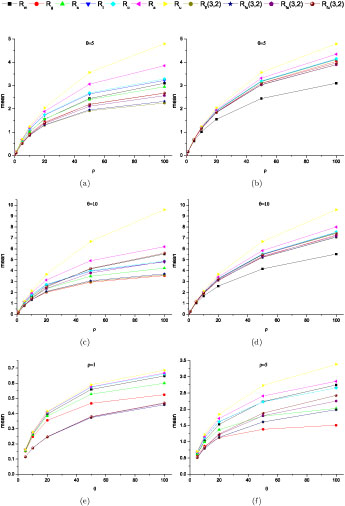
**Performance comparison of local bounds (a, c, e, f) and composite bounds (b, d) under the infinite site model (n = 10)**. (a): local bounds, *θ* (a): local bounds, *θ = 5.* (b): composite bounds, *θ = 5.* (c): local bounds, *θ =* 10. (d): composite bounds, *6θ=* 10. (e): local bounds, *ρ =* 1. (f): local bounds, *ρ =* 5.

**Figure 6:**
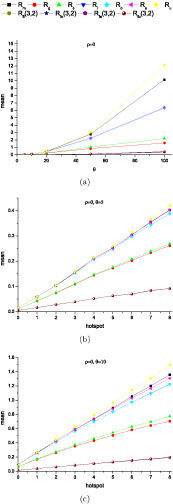
Effects of high mutation rates (a) and mutation hot-spots with *θ =* 5 (b) or *θ =* 10 (c) *(ρ = 0, n=* 10)

Figure 5(a)–5(d) compare the means of several lower bounds, *R_m_, R_g_, R_s_, R_I_, R_o_, R_a_* and an upper bound *R_u_* with increasing *ρ (θ =* 5 and 10) under the infinite site model. *R_fi_* (3, 2), *R_fo_* (3, 2), *R_fa_* (3, 2) and *R_fu_* (3, 2) were also computed and compared with the same simulated data. These results showed that *R_fi_* (3, 2), *R_fo_* (3, 2), *R_fa_* (3, 2) and *R_fu_* (3, 2) were slightly conservative (but still informative) under the infinite site model. For all bounds except *R_m_*, composite bounds were better than the corresponding local bounds and a better local bound always led to a better composite bound. As to all the composite bounds, the ranks of performance were *R_a_ ≥ R_o_ ≥ R_I_ ≥ R_s_ ≥ R_g_ ≥ R_m_* in most cases. The differences between *R_o_, R_I_* and *R_s_* were small. *R_o_* had the same computational efficiency as *R_I_* but with a slightly improved estimation. If *θ* and *ρ* were not very large, at most of the time, the difference between *R_a_* and *R_u_* was quite small. Since *R_a_* and *R_u_* are lower and upper bounds of *R_min_*, *R_a_ = R_u_* means *R_min_* is found. Even when they are not equal, if their difference is small, we can still obtain an informative interval where *R_min_* is located. Figure 5(e) and 5(f) show the increase of the means of local bounds with increasing *θ* and relative small *ρ.* Obviously, increasing *θ* will produce more polymorphic sites in DNA samples and increase the power to detect ancient recombination events. But the results showed that the power increase became slower when *θ* >> *ρ* due to the fact that the limit of the lower bounds is determined by *R_min_.* Figure 6(a) shows the increase of local bounds with the increase of *θ* without recombination *(ρ =* 0) under the finite site model. The results can be summarized as follows. Even with *ρ =* 0, the increased number of recurrent mutations with the increase of *θ* produced false positive signals of recombination events. All the bounds assuming the infinite site model were not robust to recurrent mutations, especially *R_u_* and *R_m_.* On the other hand, the bounds with *c_m_* = 3 and *c_r_* = 2 showed good robustness to recurrent mutations. Figure 6(b) and 6(c) show the effects of mutation hot-spots on the local bounds with *ρ =* 0. A mutation hot-spot was simulated by randomly superimposing a site with a 100 fold mutation rate per site as that of the sequence on average. The *θs* shown in Figure 6(b) and 6(c) were those of the sequences before superimposing hot-spots. Again, the bounds with *c_m_* = 3 and *c_r_* = 2 were more robust to mutation hot-spots than those assuming the infinite site model.

### Examples

#### Recombination analysis of the Adh gene locus

Kreitman [17] sequenced 11 *Drosophila melanogaster* alcohol dehydrogenase (Adh) genes from five natural populations and found 43 SNPs excluding insertion/deletions. This data set has become a benchmark for recombination analysis. Song and Hein [6,18] concluded that the exact number of *R_min_* equals seven. We applied the upper and lower bounds to this data set with or without extension to allow for recurrent mutations.

The results (Table 1) showed that under the infinite site model, the composite bounds of *R_I_, R_o_, R_a_* and *R_u_* all equal seven. To be more conservative and consider the effects of recurrent mutations, we manipulated the costs of recurrent mutations and recombinations such as those shown in Table 1, which allow for one, two, three and four continuous recurrent mutations. The results of *R_fo_* (*c_m_*, *c_r_*) and *R_fu_* (c_m_, c_r_) suggested that the same data could also be explained by three or four recombinations with two recurrent mutations, or one recombination with eight recurrent mutations, or 11 recurrent mutations exclusively.

**Table 1:** Local and composite bounds for the Adh data set.

*c_m_*	*c_r_*	*N_m_*	*R_m_*	*R_g_*	*R_s_*	*R_I_*	*R_o_*	*R_a_*	*R_u_*
∞	1	0	5[5]	2[6]	3[6]	4[7]	4[7]	5[7]	7
3	2	1				3	3(2)	4	4(2)
3	4	2				1	1(8)	0	1(8)
3	5	3				1	1(8)	0	1(8)
3	7	4				0	0(11)	0	0(11)

*c_m_* = ∞ and *c_r_* = 1 corresponds to the infinite site model. *N_m_* stands for the number of continuous recurrent mutations allowed. The numbers outside the brackets are local bounds. The numbers in square brackets are composite bounds. The numbers in round brackets are numbers of recurrent mutations associated with the corresponding number of recombinations.

#### Recombination analysis of the human LPL locus

Nickerson et al. [19] sequenced a 9.7 kb genomic DNA from the human lipoprotein lipase (LPL) gene with a total of 142 chromosomes from three populations (Jackson, North Karelia and Rochester). The amount of recombination detectable in this data was previously analyzed by Clark et al. [20] and then by Templeton et al. [21]. However, the conclusions drawn from these two studies were quite different. Templeton et al. [21] used a parsimony-based method to infer the minimum number of recombinations and found 29 recombination events clustering approximately at the center region of the sequence. They suggested this could be due to an elevated rate of recombination at that region. But Clark et al. [20] applied *R_m_* to the data and found no strong clustering of recombinations, which can be explained by false positives caused by recurrent mutations [21] or lack of power [7]. With the development of new methods for lower bounds, this data has been analyzed by different authors in recent years. Some [11] supported the clustering of recombinations while others [7,8] did not.

**Figure 7:**
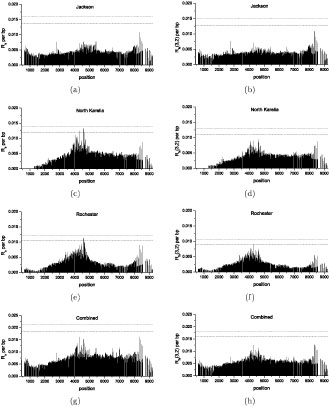
**Distribution of *R_a_* (a, c, e, g) and *R_fa_* (3, 2) (b, d, f, h) per bp along LPL haplotypes**. (a): Jackson population, *R_a_.* (b): Jackson population, *R_fa_* (3, 2). (c): North Karelia population, *R_a_*. (d): North Karelia population, *R_f_*_a_ (3, 2). (e): Rochester population, *R_a_*. (f): Rochester population, *R_fa_* (3, 2). (g): combined population, *R_a_*. (h): combined population, *R_fa_* (3,2). Dashed line and dotted line represent 95% and 99% significance level, respectively.

We applied *R_a_* and *R_fa_* (3, 2) to the data with all insertion/deletions removed. In detail, first we calculated the local bounds of *R_a_* and *R_fa_* (3, 2) for all continuous subsets of polymorphic loci that can distinguish less than or equal to 15 distinctive haplotypes in the data. Then approximate composite bounds (see **Discussion**) of *R_a_* and *R_fa_* (3, 2) were calculated. For each pair of loci if their distance is larger than 500bp but less than 5kb, the estimated number of recombination events was divided by the distance and recorded as an estimation of the *R_a_* or *R_fa_* (3, 2) per bp, which is shown in Figure 7 as a histogram at the center of that region. Similar procedures have shown to be successful in discovering the true positions of recombination hot-spots [11].

To test the significance of possible recombination hot-spots, we used simulation to determine the significance level of the maximum of *R_a_* or *R_fa_* (3, 2) per bp. We assumed that *R_a_* or *R_fa_* (3, 2) per bp follows a Poisson distribution with a mean estimated from the *R_a_* or *R_fa_* (3, 2) of the whole gene. Then we simulated *R_a_* or *R_fa_* (3, 2) for each pair of continuous loci and calculated the average *R_a_* or *R_fa_* (3, 2) per bp for each pair of loci that with a distance between 500bp and 5kb. This procedure was replicated 10,000 times and the empirical distribution of the maximum of *R_a_* or *R_fa_* (3, 2) per bp was obtained. Figure 7 (a, c, e, g) shows that *R_a_* per bp increased at the center of the sequences in the North Karelia and Rochester populations (significant at the 95% level), but this trend was less obvious (statistically not significant) in the Jackson population or the combined population. We used *R_fa_* (3, 2) instead of *R_a_* to make a conservative measure of the amount of recombinations. The pattern remained but the high peaks of *R_fa_* (3, 2) in North Karelia population and Rochester population were no longer statistically significant (Figure 7 (b, d, f, h)). This result suggested that those possible false positives produced by recurrent mutations may indeed cause the clustering pattern, other than disperse it.

## Discussion

Although the dynamic programming algorithm used in *R_s_, R_I_*, *R_o_, R_a_* and *R_u_* is a significant improvement over the original algorithm proposed by Myers and Griffiths [7], it can be quite slow when the number of haplotypes is large. Alternatively, we can use a heuristic search algorithm to approximate the local bound. Random-restart hill-climbing is a widely used heuristic search algorithm in artificial intelligence [22]. The basic idea of hill-climbing is as follows. We begin with a random order of the sequences, then we compute a local bound *R (R_s_, R_I_, R_o_, R_a_* or *R_u_)* with this fixed order such as Algorithm A.2 or A.3. Record it as *R_old_.* Then we randomly replace the positions of two sequences (a flip) to form a new order and compute *R* with the new order again. Repeat *k* times and we take the minimum of these *k* new estimations of *R* as *R_new._* If *R_new_* ≥ *R_old_,* stop. Otherwise, replace *R_old_* with *R_new_* and begin another round of *k* flips from the new order that produced *R_new_.* Repeat this procedure until *R_new_ ≥ R_old_*. Then this *R_old_* is an approximation of *R* with dynamic programming. Then we restart the hill-climbing with another random order and repeat *m* times. The minimum of all estimations is taken as a result. Note that the heuristic approximation of *R_u_* is still a valid upper bound, but that of any lower bound may not be a valid lower bound.

Other than using the heuristic search algorithm described above to approximate local bound, we can also approximate the composite bound, e.g. only the local bounds on all continuous regions with *m* or less sites are computed and used to estimate the composite bound. With the limit of sites, the number of haplotypes for the local bounds is also limited so that it prevents the need for large computational complexity. Alternatively, one can directly set a limit on the number of haplotypes used to compute the local bounds. The rational behind this procedure is that the information of the local recombination event between two sites *s_l_* and *s*_*l*+1_ is mostly contained in sites that are closely linked to them. The sites far away from *s*_*l*_ and *s*_*l*+1_ contain little information so that adding those sites has little contribution to the composite bound.

## Conclusions

In summary, the contributions of this research are several algorithms for estimating the lower bound of the minimum number of recombination events in the history of a sample. These new lower bounds are shown to be better than existing ones under the infinite site model. Furthermore, they are extended to allow for recurrent mutations, which are robust to high mutation rates and mutation hot-spots. These extended bounds can be used as a conservative measure of the amount of recombination or can be used to show different combinations of recombination and recurrent mutations that can produce the same polymorphic pattern in the sample.

## List of abbreviations used

ARG: ancestral recombination graph

Adh: alcohol dehydrogenase

LPL: lipoprotein lipase

## Competing interests

The authors declare that they have no competing interests.

## Authors contributions

XL participated in the design of the study, carried out the algorithm development and testing, and drafted the manuscript. YF conceived of the study, participated in its design and helped to draft the manuscript.

All authors read and approved the final manuscript.

## Appendices A: Algorithms

**Algorithm A.1** An algorithm for computing *R_u_* with fixed order

**Compute**_R_M_ with fixed order

input: Set *M* of all sequences

return: *R_u_*

local variable:

*n*: number of sequences in *M*

*m*: a subset of *M*

*m_−j_*: a subset of *m* by removing sequence *j*

for *i =* 1 to 3

subset *m* =first *i* sequences of *M*

*R_u_* [*m*] = 0

for *i =* 4 to *n*

subset *m* =first *i* sequences of *M*

if sequence *i* is redundant

*R_u_* [*m*] = *R_u_*[*m_−i_*]

else

*R_u_* [*m*] = *I_i_* [*m_−i_*] + *R_u_* [*m_−i_*]

return *R_u_* [*M*]

**Algorithm A.2** An algorithm for computing *R_I_* or *R_o_* with fixed order

**Compute**_*R_I_* or *R_o_* with fixed order

input: Set *M* of all sequences

return: R_I_

local variable:

*n*: number of sequences in *M*

*m*: a subset of *M*

*m_−j_*: a subset of *m* by removing sequence *j*

for *i =* 1 to 3

subset *m* =first *i* sequences of *M*

*R_d_*[*m*]=0, *R_I_*[*m*]=0

for *i =* 4 to *n*

subset *m* =first *i* sequences of *M*

if sequence *i* is redundant

*R_d_* [*m*] = *R_d_* [*m*_−*i*_]

*R_I_* [*m*] = *R_I_* [*m_−j_*]

else

*R_d_* [*m*] = A [*m_−l_*] + *R_d_* [*m_−i_*]

*R_I_* [*m*] = max{l + *R_I_* [*m_−i_*], *R_d_* [*m_−i_*] + *I_i_* [*m_−i_*]}

return *R_I_* [*M*]

**Algorithm A.3** An algorithm for computing *R_a_* with fixed order

**Compute**_R_a_ with fixed order

input: Set *M* of all sequences

return: *R_a_*

local variable:

*n*: number of sequences in *M*

*m*: a subset of *M*

*m*′: an augmented sequence set of *m*

*m__j_*: a subset of *m* by removing sequence *j*

*p_j_*: hypothetical parent sequence of sequence *j*

for *i =* 1 to 3

subset *m* =first *i* sequences of *M*

*m′ =ø, R_d_*[*m*] = 0, *R_a_*[*m*] = 0

for *i =* 4 to *n*

subset *m* =first *i* sequences of *M*

if sequence *i* is redundant in *m ∪ m′*

*m′ = m′_−i_*, *R_d_* [*m*] = *R_d_* [*m_−i_*], *R_a_* [*m*] = *R_a_* [*m_−i_*]

else

$$\begin{array}{l} R_{a}\;\left[m\right]\;=\;\max\;\left\{\text{1}\;+\;R_{a}\;\left[m_{-i}\right],\;R_{d}\left[m_{-i}\right]\;+\;I_{i}\;\left[m_{-i}\;\cup \;{m^{\prime }}_{-i}\right]\right\}\\ R_{d}\left[m\right]\;=\;1\;+\;R_{d}\;\left[m_{-i}\right] \end{array}$$

if sequence *i* is an indirect witness

*m′ = m′* ∪ *p_i_*

return *R_a_* [*M*]

## Appendix B: Proof of *R_a_* as a lower bound

Here we present a simple proof for Algorithm A.3 as a valid lower bound. Bafna and Bansal [11] has proved that *R_I_* is a valid lower bound of *R_min_* given a particular order of the sequences. This conclusion is true not only when all recombination intermediates are unknown, but also in the case if some “true” recombination intermediates are recovered in the order. If an indirect witness *j* introduces exactly one mutation into sequence set *m,* then forming a *p_j_* (the hypothetical parent sequence of *j*) by replacing the mutant allele with the “wild-type” allele of that site will recover the last recombination intermediate **(LRI)** that leads to *j* via one mutation. For example, in Figure 2(a), the LRI of indirect witness 11111 is 10111. If an indirect witness *j* introduces *n (n* ≥ 2) mutations into sequence set *m*, there are multiple possible LRIs of *j* but only one of them is the “true” LRI. However, if we form a *p_j_* by replacing the alleles on the mutant sites of the true LRI with missing data, *I_j_*[*m_-__j_ ∪ p_j_*] must be less than or equal to *I_j_*[*m_−j_* ∪ true LRI of *_j_*], since in calculating *I*[*c, h*] a missing data is never regarded as different to any alleles. Similarly, *I_k_*[*m_−j_* ∪ *p_j_* ∪ *S_k_*] must be less than or equal to *I_k_*[*m_−j_* ∪ true LRI of *j* ∪ *S_k_*], where *k* is a possible offspring of *j* and *S_k_* is a set of other possible parent sequences of *k.* So that, by augmenting the *p_j_* and then follow the procedure of *R_I_* we can get an estimation less than or equal to that with augmenting true LRIs. Then the procedure *(R_a_)* must produce a valid lower bound.
